# Editorial: Scientific Rigor or Rigor Mortis?

**DOI:** 10.1523/ENEURO.0176-16.2016

**Published:** 2016-07-07

**Authors:** Christophe Bernard

A large number of editorials/articles have been published recently on scientific rigor, both in the general media and scientific press. *eNeuro* is joining this conversation with two commentaries, the first published today by Dr. Oswald Steward, University of California, Irvine, and the second to be published July 14 by Dr. Kate Button, University of Bath. The public, and more surprisingly scientists themselves, appear to be discussing scientific rigor as a novel problem. It is not. Issues of scientific rigor have always been there, from the first time humans tried to interpret the observations they were making by proposing conceptual frameworks and testing theories.

Take, as an example, the fight between Louis Pasteur and Claude Bernard in the nineteenth century on the mechanisms underlying fermentation. Claude Bernard, who defined the Scientific Method, claimed that fermentation could occur without the presence of microorganisms, in striking opposition to Pasteur’s theory. After Bernard’s death, Pasteur published a rebuttal of Bernard’s results, stating that Bernard had lacked scientific rigor when doing the experiments on fermentation.

It is surprising that Claude Bernard could make fundamental errors of experimental design, because he had written about what the proper behavior of a scientist should be. In fact, what he wrote may be interpreted as the essence of scientific rigor: “*The experimental method is nothing but bringing observation and experiment into operation in order to get access to scientific truth. Some use the results of observation and experiment to build theories that they no longer put to test. … Instead, one’s inferences are to be tested by new experiments. … However, this is not yet sufficient. Even when attempting to verify one’s inference by an experiment or an observation, it is necessary to remain the slave of the observation, as well as of the experiment. One must not be overcome by one’s inductive idea that is nothing but a hypothesis. I can say that I follow such a precept. Thus, the verification of my inferring hypothesis, whatever its likelihood, does not blind me. I hold conditionally to it. Therefore, I am trying as much to invalidate as to verify my hypothesis. In short, I do research with an open mind. This is the reason why I so often found results I was not looking for while investigating other things I could not find. The truth must be the goal of our studies. Being satisfied by plausibility or likelihood is the true pitfall*.”

Why did the apostle of scientific rigor fail to apply the rules he defined himself? His fight with Pasteur? Ego? The conviction that he was right? The history of science is full of studies designed to prove/disprove theories without appropriate controls. Interestingly, after Pasteur’s death, the dispute was resolved; a disciple commenting about the controversy said, “One was not wrong, and one was right.” As sometimes happens in science, Bernard’s intuition was correct, although the experiments were not adequately performed to support his theory. However, in many instances, the lack of rigor leads to results that cannot be reproduced (it is estimated that 30% of the papers published by the two main science magazines cannot be reproduced, and 30% of the results are partially reproducible).

Our motivation to publish often leads us to neglect scientific rigor. In a SfN webinar on “Minimizing Bias in Experimental Design & Execution”, I mentioned the example of authors who were pressed to perform a pharmacological test in animals to get their paper published in *Nature Medicine*. They did the experiment with the minimum number of animals (*n*=5) that would satisfy reviewers. These preclinical results were taken as solid, leading to clinical trials, which were stopped because of negative/deleterious results.

Is there someone to blame for this loss of time and resources? I think that the fault lies with the system itself. In a “publish or perish” scientific world, obtaining a PhD, getting a research position, and grants depend upon on our publications and the diktat of the impact and H factors. Because competition is fierce and because positions/resources are limited, we need to arrive first. This does not mean that we consciously forget about caution and rigor. Most likely it is unconscious as we tend to privilege the instant, instead of taking the time to pause and think. The present state of the scientific world naturally emerged from our own behavior and from the set of rules imposed by funding agencies and universities. If no specific individual or stakeholder is to blame, realizing the nature of the problem should entice us to discuss it. This may be for a different series of commentaries. The present goal is to address another key aspect of scientific rigor, an issue on which we can directly intervene, ie the way we do and evaluate science, which is often done without clear guidelines, and more importantly, without proper training. Are there solutions to this problem?

The first step is to recognize the nature and limitations of what we are doing. Studies are performed with increasingly sophisticated and complex instruments. Because different laboratories do not use the same systems in a similar manner, we end up with different observations, and perhaps, opposing interpretations. In this context, scientific rigor is attempting to do the job as well as one can, ie, to limit as best as we can the intrinsic caveats and pitfalls of the experimental approach.

The second step is to adopt a common conceptual framework to do and interpret experiments. When we design experiments, we often follow protocols that are commonly used in the field. When we review papers and grants, we check whether common procedures are being followed. But this “street knowledge” is not scientifically grounded. Consider the number of papers published in the two main science magazines with *n*=3–5 (I have even seen *n*=1) experiments. Following Claude Bernard’s terminology, these numbers likely reflect scientific plausibility or likelihood, not truth.

Yet, it is not always possible to conduct experiments to reach rigorous statistical significance (eg, if several months of hard work are required for each +1 increment of the “*n*” value); however, underpowered papers still have an important function, as they can provide hypotheses to drive a field in new directions. Crucially, in this type of exploratory study, authors should clearly state that their results should be confirmed and new experiments performed. Currently, many of the results that come from a first observation of a new phenomenon are taken for granted, and can become dogma. Going against dogma is difficult, and whole fields can go astray for extended time periods as others try to replicate high-profile findings.

Scientific rigor is not always taught at the Masters/PhD level. Hence, when we start to perform experiments, we may adopt common laboratory practices, sometimes without questioning them, and which do not meet rigorous experimental criteria. We may then go on to reproduce those faulty practices in our own laboratory.

One of the missions of *eNeuro* is to provide the scientific community with teaching and training elements. For example, we have started to address the issue of how to peer review a manuscript with the first in a series of webinars “Tricks of the Trade: How to Peer Review a Manuscript”. I am now proud to introduce a series of commentaries on the issue of scientific rigor: “A Rhumba of R’s; Replication, Reproducibility, Rigor, Robustness: What Does a Failure to Replicate Mean?” and “Statistical Rigor and the Perils of Chance”. Some concepts developed in these papers may appear to be common sense, which they are, but it is important to stress that by following simple guidelines, one can easily avoid some pitfalls of the scientific approach. We talk about the importance of taking into account chance findings, false-negatives, what statistical analysis really means, etc. We hope to identify as many issues as possible that can make our scientific approach more rigorous and our interpretations more accurate. If you are interested in contributing to this series of commentaries, do not hesitate to contact me at eNeuroeditor@sfn.org.

